# The usefulness and feasibility of a screening instrument to identify psychosocial problems in patients receiving curative radiotherapy: a process evaluation

**DOI:** 10.1186/1471-2407-11-479

**Published:** 2011-11-08

**Authors:** Anna PBM Braeken, Gertrudis IJM Kempen, Daniëlle Eekers, Francis CJM van Gils, Ruud MA Houben, Lilian Lechner

**Affiliations:** 1 Maastricht University, Faculty of Health, Medicine and Life Sciences, Department of Health Services Research, School for Public Health and Primary Care (CAPHRI), Maastricht, the Netherlands; 2 Netherlands Open University, Faculty of Psychology, Heerlen, the Netherlands; 3 Dept. of Radiation Oncology (MAASTRO), GROW - School for Oncology and Developmental Biology, Maastricht University Medical Centre, the Netherlands; 4 Institute Verbeeten, Radiation Oncology, Tilburg, the Netherlands

## Abstract

**Background:**

Psychosocial problems in cancer patients are often unrecognized and untreated due to the low awareness of the existence of these problems or pressures of time. The awareness of the need to identify psychosocial problems in cancer patients is growing and has affected the development of screening instruments. This study explored the usefulness and feasibility of using a screening instrument (SIPP: Screening Inventory of Psychosocial Problems) to identify psychosocial problems in cancer patients receiving curative radiotherapy treatment (RT).

**Methods:**

The study was conducted in a radiation oncology department in the Netherlands. Several methods were used to document the usefulness and feasibility of the SIPP. Data were collected using self-report questionnaires completed by seven radiotherapists and 268 cancer patients.

**Results:**

Regarding the screening procedure 33 patients were offered to consult a psychosocial care provider (e.g. social worker, psychologist) during the first consultation with their radiotherapist. Of these patients, 31 patients suffered from at least sub-clinical symptoms and two patients hardly suffered from any symptoms. Patients' acceptance rate 63.6% (21/33) was high. Patients were positive about the content of the SIPP (mean scores vary from 8.00 to 8.88, out of a range between 0 and 10) and about the importance of discussing items of the SIPP with their radiotherapist (mean score = 7.42). Radiotherapists' perspectives about the contribution of the SIPP to discuss the different psychosocial problems were mixed (mean scores varied from 3.17 to 4.67). Patients were more positive about discussing items of the SIPP if the radiotherapists had positive attitudes towards screening and discussing psychosocial problems.

**Conclusions:**

The screening procedure appeared to be feasible in a radiotherapy department. In general, patients' perspectives were at least moderate. Radiotherapists considered the usefulness and feasibility of the SIPP generally to be lower, but their evaluations were mixed. A positive attitude to using screening instruments like the SIPP needs to be encouraged among radiotherapists, as this may not only improve the usefulness of a screening instrument, but also patients' satisfaction with care.

**Trial Registration:**

ClinicalTrials.gov: NCT00859768

## Background

Cancer, as well as its sometimes invasive and aggressive treatment, has a great impact on a patient's life. Previous studies have highlighted that cancer patients undergoing radiotherapy treatment (RT) may experience psychosocial problems such as symptoms of depression and anxiety [1,2], which may negatively affect health and treatment-related outcomes [3]. It is important to detect psychosocial problems at an early stage because treatment of sub-clinical symptoms of psychosocial problems may prevent further deterioration in the patient and the development of psychiatric co-morbidity [4]. Yet, psychosocial problems are often unrecognized and untreated due to the low awareness of the existence of psychosocial problems or pressures of time [5,6]. Furthermore, physicians are more focused on physical symptoms [7-9], and may feel more able to help with physical problems than with emotional ones [10]. Another aspect is that cancer patients do not tend to report psychological problems to their physician; some patients may regard psychosocial care as stigmatizing and on this basis be reluctant to seek help [6,11].

Awareness of the need for identifying psychosocial problems is growing, resulting in the development of screening instruments [12]. Several studies examined the use of screening instruments [12-16]. Most studies reported that using these instruments gave better insight into patients' psychosocial problems and facilitated patient-physician communication on the topics of the instrument [7,12,17]. Our study, a randomized controlled trial recently showed that using a simple screening instrument in a radiation oncology department can be valuable in timely treatment of these problems but, however, no significant effects were observed for the number of referred patients, nor for improvement of the patients-radiotherapist communication (paper submitted, article available on request).

In parallel to this trial, a process evaluation was carried out. The purpose of this process evaluation was to evaluate the feasibility and usefulness of this screening instrument (SIPP: Screening Inventory of Psychosocial Problems) and to identify factors that may explain the lack of effectiveness on referral patterns and patients-radiotherapist communication in patients receiving curative RT. The specific aims were:

1. To gain insight into the procedure of using the SIPP in a radiation oncology department.

2. To investigate the degree to which the SIPP was considered useful and feasible by both radiotherapists and cancer patients. Feasibility, including acceptability, of screening instruments is an important aspect that determines successful implementation of such tools in radiation oncology departments [18,19].

To investigate whether perspectives of patients and radiotherapists on the usefulness and feasibility of the SIPP are associated. We hypothesized that more positive attitudes of radiotherapists towards discussing psychosocial problems in daily practice will result in more positive perspectives on the usefulness of the SIPP among patients. Previous studies have reported that the way physicians address psychosocial problems affects how patients perceive the interactions with their physician [20,21].

## Methods

### Study design and participants

This process evaluation study, part of a larger clustered randomized controlled trial [22], used several methods to document the usefulness and feasibility of the SIPP, a screening instrument to identify different psychosocial problems in cancer patients (see Appendix A). The study was conducted between April 2008 and October 2009 at Institute Verbeeten (BVI), a radiation oncology department in the city of Tilburg in the south of the Netherlands. Patients with the following characteristics were included: cancer diagnosis of lung, prostate, bladder, rectum, breast, cervix, skin, endometrial or non-Hodgkin lymphoma; age over 18 years; patients without metastases; and able to provide written informed consent. Exclusion criteria were: receiving palliative treatment; receiving ≤ 10 fractions of radiotherapy treatment; unable to read and speak Dutch and unable to complete questionnaires (e.g., too sick). Seven radiotherapists working at BVI were randomly included in this study. Patients were linked to their radiotherapist. This study was approved by the Medical Ethics Committee of the Twee Steden Hospital in Tilburg, the Netherlands.

### Intervention

The SIPP was chosen as the intervention screening instrument because it is a Dutch, simple screening instrument designed to identify multiple aspects of psychosocial problems in cancer patients [23]. Furthermore, it is used in several hospitals and studies [14,24-26]. Psychometric properties of the SIPP were studied recently and found acceptable [23]. The SIPP comprises 24 items and assesses physical complaints (seven items, Cronbach's alpha = 0.76), psychological complaints (10 items, Cronbach's alpha = 0.89), social problems (four items, Cronbach's alpha = 0.56), and sexual problems (three items, Cronbach's alpha = 0.51). Items are rated on a three-point scale of "0" (No), "1" (Sometimes) and "2" (Yes) with an additional option of "Not Applicable" (score 0) for the sexual problems subscale (for items see Appendix A). Higher scores indicate higher levels of psychosocial problems. Prevalence rates of patients with at least sub-clinical and clinical symptoms of psychosocial problems were assessed with the SIPP by using suitable cut-off scores (see footnote Table 1) [23].

**Table 1:** Patients' extent of psychosocial problems and amount of referred patients as assessed with the SIPP

	SIPP before first consultation (n = 263)		SIPP before consultation at the end of treatment (n = 250)				SIPP before first consultation		SIPP before consultation at the end of treatment		SIPP before first consultation		SIPP before consultation at the end of treatment	
	
	Mean	(SD)	Mean	(SD)	*P*-value		At least sub- clinical symptoms n (%)^b^	Referred n (%)	At least sub- clinical symptoms n (%)^b^	Referred n (%)	Clinical symptoms n (%)^c^	Referred n (%)	Clinical symptoms n (%)^c^	Referred n (%)
Physical complaints (score range 0-14)	3.8	(3.1)	4.7	(3.2)	0.00*		93 (34.7)	21 (22.5)	113 (42.2)	7 (6.2)	69 (25.7)	16 (23.2)	92 (34.3)	7 (7.6)
Psychological complaints (score range 0-20)	5.1	(4.4)	3.9	(4.3)	0.00*		101 (37.7)	29 (28.7)	67 (25.0)	8 (11.9)	41 (15.3)	20 (48.8)	31 (11.6)	6 (19.4)
Social problems (score range 0-8)	0.6	(1.1)	0.5	(1.0)	0.01*		52 (19.4)	17 (32.7)	23 (8.6)	5 (21.7)	12 (4.5)	8 (66.7)	6 (2.2)	1 (16.7)
Sexual problems^a^ (score range 0-6)	0.8	(1.3)	0.7	(1.2)	0.36		10 (3.7)	3 (30.0)	4 (1.5)	0 (0.0)	5 (1.9)	3 (60.0)	2 (0.7)	0 (0.0)
Total score (score range 0-24)	10.4	(7.3)	9.8	(7.2)	0.18		149 (55.6)	31 (20.8)	126 (47.0)	9 (7.1)	93 (34.7)	28 (30.1)	97 (36.2)	9 (9.3)

SD: Standard deviation

*The level of statistic significance is 0.05 (two-tailed). A Holm-bonferroni correction was applied for multiple comparisons.

a In order to calculate the mean score of sexual problems, the answer category "Not Applicable" was scored as a missing value.

Cut-off scores:

b An indication for at least sub-clinical symptoms (at least moderate) of psychosocial problems is to fulfill to one of more of the cut-off scores as described below:

1. Cut-off score of ≥ 5 on the physical complaints subscale

2. Cut-off score of ≥ 6 on the psychological complaints subscale

3. Cut-off score of ≥ 1 on the item: "Would you like to discuss these problems with someone" on the social/financial problems subscale

4. Cut-off score of ≥ 1 on the item: "Would you like to discuss these problems with someone" on the sexual problems subscale

cAn indication for clinical symptoms (high) of psychosocial problems is to fulfill to one of more of the cut-off scores as described below:

1. Cut-off score of ≥ 6 on the physical complaints subscale

2. Cut-off score of ≥ 10 on the psychological complaints subscale

3. Cut-off score of 2 on the item: "Would you like to discuss these problems with someone" on the social/financial problems subscale

4. Cut-off score of 2 on the item: "Would you like to discuss these problems with someone" on the sexual problems subscale

Patients received the SIPP before the first consultation with their radiotherapist and before the consultation at the end of their RT period since these time points were considered relevant for psychosocial support [27]. At both time points, the completed SIPP was handed to the radiotherapist at the start of the consultation. Radiotherapists had to check and discuss the scores of the SIPP to get an impression of potential psychosocial problems and patients' needs for psychosocial care. Radiotherapists were asked to indicate on the SIPP whether patients were offered an appointment with a psychosocial care provider (e.g. psychologist, social worker, physician or nurse) and whether patients accepted their offer.

The study protocol was explained to the radiotherapists and other involved personnel at BVI (e.g. physician-assistants) [22]. Radiotherapists were trained in using and interpreting the SIPP, including interpretation of scores and the type of potential psychosocial problems and the need for psychosocial care during a one-hour training session. Training was given by the researcher and two social workers. Suitable cut-off scores for symptoms were explained to radiotherapists. A manual was prepared with interpretation of the scores.

### Measurement

#### Baseline characteristics of the patients and radiotherapists

Socio-demographic variables were assessed directly after the first consultation (Table 2). Medical status before RT included cancer site, tumour classification (TNM) and the Karnofsky Performance Index. Variables were extracted from patients' medical records. Socio-demographic variables of the radiotherapists such as age, sex and number of years of work experience were obtained via personnel records of BVI.

**Table 2:** Characteristics of the patients (n = 268)

Variables		n	%
Age (years)			
Mean (SD)	62.4 (10.8)		
Range	30.0-88.0		
Sex			
Female		183	68.3
Marital status			
Married/living together		207	77.2
Unknown		5	1.9
Educational level			
Elementary		103	38.4
High school		116	43.3
Higher Education/University		43	16.0
Unknown		6	2.2
Diagnosis			
Prostate/Bladder		50	18.7
Lung		21	7.8
Breast		145	54.1
Cervix/Endometrial		9	3.4
Rectum		40	14.9
Non-Hodgkin Lymphoma		3	1.1
T-status (size of the primary tumor)			
Tin-situ		7	2.6
T1/T2		152	56.7
T3/T4		58	21.6
Unknown		51	19.0
N-status (degree of spread lymph nodes)			
N0		141	52.6
N1/N2		64	23.9
N3/N4		5	1.9
Unknown		58	21.6
M-status (presence of metastasis)			
M0		203	75.7
Unknown		65	24.3
Karnofsky Performance Index^a^			
100/90		194	72.4
80/70		24	8.9
Unknown		50	18.7
Chemo therapy			
Before radiotherapy treatment		66	24.6
During radiotherapy treatment		25	9.3
Time frame between start and end of the radiotherapy treatment (days)			
Mean (SD)	38 (6.7)		
Median	37		
Range	(16-65)		

SD: Standard deviation

a Score range 0-100, higher score indicates better physical functioning

#### Questionnaire to assess patients' perspectives

**Figure 1:**
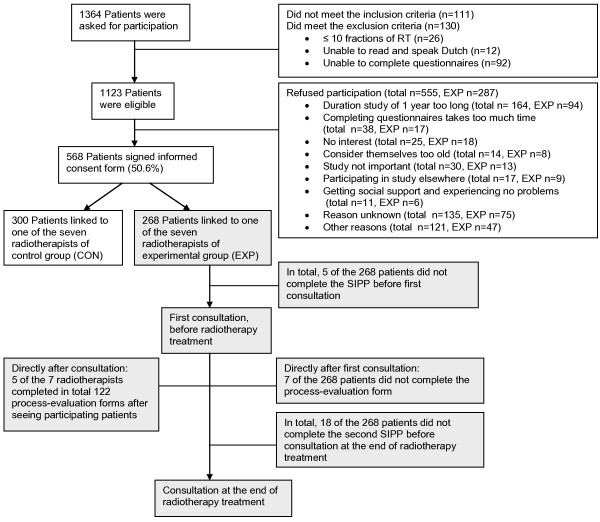
**CONSORT flow diagram of the patients' inclusion procedure and data collection process evaluation (grey blocks)**.

Data on patients' perspectives of the usefulness and feasibility of the SIPP were collected using a self-report questionnaire. The questionnaire was completed directly after the first consultation (see Figure 1). This questionnaire included: one open-ended question about the time (in minutes) taken to complete the screening instrument; 11 items including written statements assessing experiences with the instrument (Table 3: items 2-12) and two open questions for suggestions for improvement of the SIPP and other remarks (Table 3: items 13-14). The 11 items were rated on a scale ranging from 0 to 10. Higher scores indicated a more positive opinion. The items 2-4 were related to the content of the SIPP (total score range 0-30, Cronbach's alpha = 0.68) and the items 5-12 concerned patients' perspectives about communication (total score range 0-80, Cronbach's alpha = 0.84).

**Table 3:** Patients' perspectives on the usefulness and feasibility of the SIPP

	Scores: theoretical range	n	Mean score (SD)	Scores: observed range	Negative perspective (%)^a^ Score ≤ 4	Moderate perspective (%)^a^ Score 5, 6	Positive perspective (%)^a^ Score ≥ 7
1. Time to complete the screening instrument	Open question (minutes)	256	5.3 (3.5)	1-20	----	----	----
**Instruments' content subscale:**							
2. Understanding the items	0(Not easy to understand)- 10(Easy to understand)	258	8.9 (1.3)	0-10	0.4	2.2	93.7
3. The instrument is pleasant to complete	0(Not pleasant to complete)- 10(Very pleasant to complete)	259	8.4 (1.5)	0-10	1.5	3.7	91.5
4. Importance of the subjects	0(Not important)- 10(Very important)	258	8.0 (1.6)	0-10	1.5	11.5	83.2
**Communication aspects subscale:**							
5. Importance of discussing the screening instrument with radiotherapist	0(Not important)- 10(Very important)	237	7.4 (2.3)	0-10	8.0	12.7	67.5
6. Physical complaints in the screening instrument were discussed with radiotherapist	0(Not discussed) - 10(Extensively discussed)	250	6.2 (3.1)	0-10	18.9	17.9	56.3
7. Psychosocial complaints in the screening instrument were discussed with radiotherapist	0(Not discussed)- 10(Extensively discussed)	249	4.6 (3.6)	0-10	37.3	16.5	39.3
8. Sexual problems in the screening instrument were discussed with radiotherapist	0(Not discussed)- 10(Extensively discussed)	241	1.6 (2.7)	0-10	73.1	7.5	9.3
9. The screening instrument was a useful tool to discuss physical complaints with radiotherapist	0(Not useful)- 10(Very useful)	246	4.1 (3.6)	0-10	43.7	17.5	30.6
10. The screening instrument was a useful tool to discuss psychosocial complaints with radiotherapist	0(Not useful)- 10(Very useful)	242	3.5 (3.6)	0-10	48.4	15.3	26.5
11. The screening instrument was a useful tool to discuss sexual problems with radiotherapist	0(Not useful)- 10(Very useful)	234	1.7 (2.8)	0-10	67.7	10.8	8.9
12. Discussing the screening instrument scores with the radiotherapist was pleasant	0(Not pleasant)- 10(Very pleasant)	223	6.4 (2.7)	0-10	11.2	24.6	47.4
**Open questions for remarks**							
13. Is there a subject that was missing from the screening instrument?	No-Yes, namely...	5	----	----	----	----	----
14. Have you any remarks?	No-Yes, namely...	0	----	----	----	----	----

SD: Standard deviation

aDue to possible missing values for several items, not all the scores add up to 100%

#### Questionnaires to assess radiotherapists' perspectives

Data on the radiotherapists' perspectives on the usefulness and feasibility of the SIPP were collected using a self-report questionnaire completed by radiotherapists directly after the first consultation with a patient. This questionnaire contained three items including written statements assessing experiences with the SIPP (Table 4: items 1-3) and one open question about the time (in minutes) that was required to discuss the SIPP with the patient (Table 4: item 4). The three items were each rated on a scale ranging from 0 to 10 (items 1-3, total score range 0-30, Cronbach's alpha = 0.89). Higher scores indicated a more positive evaluation.

**Table 4:** Radiotherapists' perspectives on the usefulness and feasibility of the SIPP

	Scores: theoretical range	n	Mean score (SD)	Scores: observed range	Negative perspective (%)^c^ Score ≤ 4	Moderate perspective (%)^c^ Score 5, 6	Positive perspective (%)^c^ Score ≥ 7
**After first consultation for each patient**							
1. The screening instrument invited to ask about the patient's psychosocial well-being	0 (Not used) - 10 (Fully used)	146	5.9 (2.8)	0-10	17.8	6.2	30.3
2. The scores gave better insight into the patient's psychosocial well being	0(Less insight) - 10(Very much insight)	146	6.5 (2.4)	0-10	12.3	4.1	36.2
3. Exchanging information about the subjects in the screening instrument gave better insight into the patient's psychosocial well being	0(Less insight) - 10(Very much insight)	146	6.3 (2.6)	0-10	16.2	4.1	34.4
4. Time required to discuss the instrument	Open question (minutes)	142	4.3 (2.5)	0-15	----	----	----
**More generally**							
5. Contribution of using the screening instrument for discussing physical complaints	0 (No contribution) - 10 (Very good contribution)	6^a^ 6^b^	3.2 (3.2) 3.5 (3.7)	0-7 0-8	66.7 66.7	0.0 0.0	33.3 33.3
6. Contribution of using the screening instrument for discussing psychosocial complaints	0 (No contribution) - 10 (Very good contribution)	6 ^a^ 6 ^b^	4.7 (3.9) 3.7 (3.9)	0-8 0-9	50.0 66.7	0.0 0.0	50.0 33.3
7. Contribution of using the screening instrument for discussing sexual problems	0(No contribution)- 10(Very good contribution)	6 ^a^ 6 ^b^	3.7 (4.0) 3.5 (4.4)	0-9 0-10	66.7 66.7	0.0 0.0	33.3 33.3
8. Usefulness of discussing (the scores on) the screening instrument	0(Not useful)- 10(Very useful)	6 ^a^ 6 ^b^	4.8 (4.0) 4.0 (4.2)	0-9 0-9	33.3 66.7	16.7 0.0	50.1 33.3
9. Discussing (the scores on) the screening instrument with the patient was pleasant	0(Not pleasant)- 10(Very pleasant)	6 ^a^ 5 ^b^	4.3 (3.5) 6.0 (4.1)	0-8 0-10	33.3 33.4	33.4 0.0	33.3 33.4
10. Contribution of discussing the screening instrument to a better quality of consultation	0(No positive contribution) - 10(Very positive contribution)	5 ^a^ 5 ^b^	5.4 (3.4) 4.4 (4.0)	0-9 0-9	40.0 50.1	0.0 0.0	60.0 33.4
11. Indication of the scores for referring patients to social caregivers	0(No good indication)- 10(Very good indication)	--- 5 ^b^	--- 4.2 (3.7)	--- 0-9	--- 24.9	--- 0.0	--- 16.6
12. Changing communication style by using the screening instrument	0(No changing)- 10(Changing)	--- 6 ^b^	--- 1.5 (2.1)	--- 0-5	--- 41.6	--- 8.3	--- 0.0
13. Feasibility of using the screening instrument during consultations for patients to bring up psychosocial problems	0(Not feasible)- 10(Feasible)	--- 6 ^b^	--- 3.8 (3.9)	--- 0-9	--- 33.3	--- 0.0	--- 16.6
**Open questions for remarks**							
14. Is there a subject that was missing from the screening instrument?	No-Yes, namely...	2	----	----	----	----	----
15. Have you any remarks?	No-Yes, namely...	2	----	----	----	----	----

SD: Standard deviation

a First measurement, 7 months after the start of the study

b Second measurement with 3 additional items (item 7-9), 13 months after the start of the study

c Due to possible missing values for several items, not all the scores add up to 100%

Seven and 13 months after the start of the study, the radiotherapists completed a questionnaire on the general usefulness and feasibility of the SIPP. This included nine items with written statements assessing experiences with the SIPP (Table 4: items 5-13) and two open questions for suggestions for improvement of the instrument or other remarks. The nine items were rated on a scale ranging from 0 to 10 (items 5-13, total score range 0-90, Cronbach's alpha = 0.98). Higher scores indicated a more positive opinion. It should be noted that the last three items (items 11-13) were measured only the second time (13 months after the start of the study).

### Data analysis

Mean differences between the scores on the first SIPP and second SIPP were analyzed by using a paired t-test. Quantitative data of the process study were analyzed by means of descriptive statistics (e.g. length of time to complete the SIPP, scores on the instruments). Qualitative data (e.g. answers to open questions) were categorized. For describing patients' and radiotherapists' different perspectives on the items of the 0-10 point scales, the scores were categorized in negative (score ≤ 4), moderate (scores of 5 and 6) and positive perspectives (score ≥ 7). We used mean scores for separate items as well as sum scores for scales across items. Associations between patients' and radiotherapists' perspectives on the usefulness and feasibility of the SIPP were analyzed with Pearson correlations. Non-parametric tests were used if data were not normally distributed. Analyses were performed with SPSS software (version 17.0; SPSS Inc., Chicago, IL). The level of statistical significance was set at 0.05 (two-tailed).

## Results

### Baseline characteristics of the patients and radiotherapists

Sociodemographic and medical characteristics of the 268 patients are listed in Table 1.

Figure 1 shows the flowchart of the number of patients in each stage of the screening procedure. In total, 23 patients did not complete the SIPP the first (n = 5) or second (n = 18) time, of which one patient did not complete the SIPP at both times. In total, 7 patients (2.6%) did not complete the process evaluation forms.

The SIPP was applied by seven radiotherapists. Three were female. The mean age was 44.6 (SD = 10.0) years (range 30 to 63 years), and on average they had worked for 14.1 years (range 4 to 35 years) as a radiotherapist.

The process evaluation forms on the usefulness and feasibility of the SIPP after each first consultation with the patient were completed by five radiotherapists for 146 patients. Two radiotherapists never completed this form. One radiotherapist never checked the SIPP for potential psychosocial problems for all his 54 participating patients. So, questions in the evaluation form after each first consultation were not applicable for him/her. The other radiotherapist reported that completing the form after each patient took too much time.

Six radiotherapists completed twice the overall (i.e. not patient-specific) process evaluation forms on the usefulness and feasibility of the SIPP. Two radiotherapists did not complete the first or second process evaluation form due to the fact that one radiotherapist had treated only one participating patient and another radiotherapist no longer worked at the BVI at about 8 months after the start of the study.

### Psychosocial problems as assessed with the SIPP

Table 2 shows patients' extent of psychosocial problems measured with the SIPP at the two time points before the consultation with the radiotherapists. Furthermore, the numbers of referred patients with symptoms of psychosocial problems to a caregiver are presented. At the end of the RT patients reported a significantly lower extent of psychological and social problems (paired t-test, *P *< 0.01 and *P *= 0.01, respectively), but significantly more physical complaints (paired t-test, *P *< 0.01) than during the first consultation. During the first consultation a total of 33 patients were offered the opportunity to consult a psychosocial care provider as a result of the screening procedure. Of these 33 patients, 31 patients suffered from at least sub-clinical symptoms, of which 28 patients suffered from clinical symptoms (Table 2) and two patients hardly suffered from any symptoms of psychosocial problems at all (not tabulated). Since 28 of the 31 patients suffered from clinical symptoms this indicates that three patients suffered only from sub-clinical symptoms. Twenty-one patients accepted the recommendation and were referred to a psychosocial care provider (not tabulated). During the consultation at the end of the RT, all nine patients who were offered the opportunity to consult a caregiver suffered from clinical symptoms (Table 2).

### Patients' perspectives on the usefulness and feasibility of the SIPP

Ratings of patients' perspectives about completing and discussing the SIPP after the first consultation are described in Table 3. On average, completing the SIPP took 5.3 minutes and 71.6% of the patients completed the SIPP in five minutes or less (not tabulated). Patients were positive about the content of the SIPP. The mean scores on the three items of the instruments' content subscale (items 2-4) varied from 8.0 to 8.9. Regarding communication aspects, most patients (67.5%) were positive about the importance of discussing items of the SIPP with their radiotherapist. However, patients were not positive about the usefulness of the SIPP to discuss psychosocial complaints and sexual problems.

### Patients' suggestions for improvement of the SIPP

Five patients made suggestions for improvement. One patient suggested adding an item on cognitive functioning, and one patient suggested adding an item about medication use. Furthermore, one patient considered it important to ask about problems at work, while another patient reported that questions on specific tumours or treatment of specific complaints were lacking. One patient would have preferred an item about receiving care at home.

### Radiotherapists' perspectives on the usefulness and feasibility of the SIPP

Ratings of radiotherapists' perspectives on using the SIPP after the first consultation are presented in the upper part of Table 4.

In 90.1% of all consultations, the time required to discuss the instrument was less than five minutes (not tabulated). With respect to specific evaluation after each consult, radiotherapists were most positive about the fact that the SIPP gave better insight into the psychosocial well-being of the patient. They were less positive about the fact that the SIPP invited them to ask about the patients' psychosocial well-being.

Radiotherapists' overall perspectives on using the SIPP were measured at two time points: about seven and 13 months after the start of the study. Their overall perspectives on the usefulness and feasibility of the SIPP was mixed (mean scores varied from 1.5 to 6.0; see lower part of Table 4). Three radiotherapists considered the SIPP to be useful and feasible. None of the radiotherapists was positive about changing their communication style by using the SIPP.

### Radiotherapists' suggestions for improvement of the SIPP

Two radiotherapists made suggestions for improvement. One preferred a tumour-specific questionnaire above a disease-specific questionnaire and another suggested an item about whether patients already received psychosocial support. Regarding feasibility, one radiotherapist suggested that physician-assistants should discuss the screening instrument with the patients.

### Associations between patients' and radiotherapists' perspectives on the usefulness of the screening instrument

Radiotherapists who considered the screening instrument a useful tool to ask about psychosocial well-being aspects (Table 4 item 8) were more likely to discuss psychosocial complaints (r = 0.2, *P *= 0.01) and sexual problems (r = 0.3, *P *< 0.01) with their patients (Table 3 items 7 and 8, respectively). Patients were more likely to report that discussing the items of the SIPP with their radiotherapist was pleasant (Table 3 item 12) if the radiotherapist considered the SIPP to be a useful tool to ask about psychosocial well-being (r = 0.2, *P *< 0.05) (Table 4 item 8).

## Discussion

This study explored the usefulness and feasibility of using a screening instrument as the SIPP in a radiation oncology department according to radiotherapists and patients. Nearly all patients completed the SIPP twice, at the beginning and at the end of RT, which is an indication of an acceptable feasibility of the screening instrument. Just as previous studies reported that not all radiotherapists were willing to use psychosocial screening instruments [19,28] in our study also one radiotherapist was not willing to use the screening instrument.

The prevalence rate of psychosocial problems among patients receiving RT with curative intent was in line with previous studies [1,29]. During the treatment trajectory patients seemed to experience different problems at different time points; the extent of psychological and social problems was lower at the end of RT, while the extent of physical complaints was higher at this time point. The latter was probably due to the high extent of side-effects at the end of RT since patients had received the maximum radiation doses. Displaying similarities with other studies [30-32], a relative small proportion of patients suffering from at least sub-clinical (7.1%-20.8%) or clinical (9.3%-30.1%) symptoms of psychosocial problems were offered the opportunity to visit a psychosocial care provider by their radiotherapist (Table 2). An explanation might be that psychosocial problems were discussed sufficiently during the consultation, which meant that receiving psychosocial support by a psychosocial care provider was not considered necessary any more [33]. Another explanation might be that according to Livingston and colleagues, physicians may be uncomfortable referring patients at a time when patients are overloaded with information (e.g. treatment, and side-effects) [34], as is the case during a first consultation. Also, physicians tend to ignore raw scores on questionnaires when they have to add them up and interpret them themselves, resulting in under diagnosis of patients suffering from psychosocial problems [35]. The latter can be prevented by using a computer-based screening instrument that forwards the screening data and presents the results directly to physicians. However, Boyes and colleagues reported that giving physicians feedback about patients' psychosocial well-being rarely contributed to physicians' decision making about patient management [28]. Furthermore, contrary to other studies [31,36-41], our results showed that when offered during the first consultation, the majority of the patients (63.6%) accepted psychosocial care. It seemed that receiving psychosocial support was acceptable for patients at that time point. A previous study reported that patients' need and acceptance for psychosocial support seemed to be related to timing [38]. Still, it should be taken into account that about one-third of the patients did not wish to be referred to a psychosocial care provider, which may imply that for some patients a barrier emerged that impeded acceptance of referral [40].

There is a discrepancy between radiotherapists' and patients' perspectives of the usefulness and feasibility of the SIPP. Patients' perspectives of the usefulness and feasibility of the SIPP were moderate to good in general. Comparable with the study of Pruyn and colleagues [25], two-thirds of the patients were very positive on the importance of discussing the screening instrument with their radiotherapist. Regarding the radiotherapists' perspectives, only a minority of radiotherapists considered the SIPP to be useful and feasible. Despite the latter, it is important to note that our outcomes showed a positive association between radiotherapists' opinion about the scores as good indicators into patients' well-being and discussing psychological problems. However, it can be vice versa: radiotherapists who traditionally more often discuss psychosocial problems were those who indicated that the screening instrument gave better insight into patients' problems. Furthermore, discussing the screening instrument was more pleasant for patients when the radiotherapists were positive about discussing it. This confirms our hypothesis that if radiotherapists have positive attitudes towards discussing psychosocial problems patients' perspectives of the usefulness of the tool will be more positive. Moreover, it also indicates the importance of patient-physician communication for high-quality care, as it may influence patients' satisfaction with care [13,20,42].

Some results of this study were remarkable. Radiotherapists were quite positive about the usefulness and feasibility of the screening instrument when they evaluated the SIPP directly after each first consultation with their patient, but more negative when the usefulness and feasibility was evaluated in general and not related to a specific consultation. A reasonable explanation is the different numbers of radiotherapists who completed the questionnaires in both situations. As reported earlier, two radiotherapists did not report their opinion after each consultation. Yet, despite the assumption that using a screening instrument may initiate better communication between patients and medical staff [7,12], none of the radiotherapists were convinced that using the screening instrument would change their communication style.

This study has several strong elements: the large number of patients that was included as well as the perspectives of both patients and radiotherapists. The study has limitations as well. First, the study was conducted in a single radiotherapy department, and although the number of patients was large, the number of radiotherapists was limited. This reduces the options for generalization of the results. Second, because the SIPP had not yet been incorporated into routine care, the radiotherapists might have gained insufficient experience to check the SIPP scores and patients' needs for psychosocial care. Partly, this could explain why radiotherapists considered the usefulness and feasibility of the screening instrument generally as low, while actual scores were mixed.

Recommendations for improving the use of a screening instrument in practice include encouraging screening at several time points as patients' needs and extent of psychosocial problems may change during the treatment trajectory. Furthermore, an important issue is to change and improve the physicians' attitudes regarding psychosocial care. This is relevant since physicians' attitudes toward discussing emotional problems may influence detection of psychosocial problems [21] and are linked with patients' satisfaction [13]. Additional skills training among physicians may be needed to improve recognition and management of psychosocial problems in cancer patients [43]. However, it could also be that nurses rather than physicians prove to be the most suitable health care professionals to discuss psychosocial problems with cancer patients. Studies reported that nurses were more willing to use screening tools than physicians [19]. Further research is necessary to optimize the use of screening instruments that may identify cancer patients' extent of psychosocial problems and improve health-related outcomes.

## Conclusion

Overall, we can conclude that the SIPP screening procedure appeared to be feasible in a radiotherapy department and that the screening procedure appears to be valued positively by most cancer patients, but not by all radiotherapists. It is useful to reflect on the fact that two-thirds of the patients rated the discussion with their radiotherapist about psychosocial problems as (highly) important. So, improving radiotherapists' attitudes towards discussing emotional problems and using screening instruments may not only improve the usefulness of a screening instrument like the SIPP, but also patients' satisfaction with care.

## Competing interests

The authors declare that they have no competing interests.

## Authors' contributions

LL and GK obtained funding together with FG, RH, DE and AB. GK, LL, FG, RH, and AB were involved in conception and design of the study. AB is the investigator and works under supervision of GK and LL. LL, GK, FG and AB designed some questionnaires. AB and DE coordinated the entry and validation of the data. AB has made substantial contribution to statistical analyses and interpretation of the data. RH was advisor in the statistical analysis plan. GK, LL, DE and RH have made contribution to interpretation of the data. AB drafted the manuscript, with input from the other authors. All authors mentioned in the manuscript read and approved the final version.

## Appendix A: Screening Inventory of Psychosocial Problems (SIPP) (Additional file 1)

## Pre-publication history

The pre-publication history for this paper can be accessed here:

http://www.biomedcentral.com/1471-2407/11/479/prepub

## Supplementary Material

Additional file 1 Screening Inventory of Psychosocial Problems (SIPP) Click here for file
