# Supplementary material for: The usefulness and feasibility of a screening instrument to identify psychosocial problems in patients receiving curative radiotherapy: a process evaluation
Source: BMC Cancer. 2011 Nov 8;11:479. doi: 10.1186/1471-2407-11-479 (PMC3247231; doi:10.1186/1471-2407-11-479)
Supplement: Additional file 1 — Screening Inventory of Psychosocial Problems (SIPP) [file 1471-2407-11-479-S1.PDF]

Screening Inventory of Psychosocial Problems (SIPP)

Having cancer may lead to various complaints. Please indicate which of the following complaints apply to you

	Yes	***Sometimes***	***No***	***N/A***
Physical complaints subscale				
1.Fatigue	☐	☐	☐	
2.Sleep disorder	☐	☐	☐	
3.Weight loss	☐	☐	☐	
4.Lack of appetite	☐	☐	☐	
5.Restriction in daily functioning	☐	☐	☐	
6.Dizziness	☐	☐	☐	
7.Pain	☐	☐	☐	
Psychological complaints subscale				
8.Worrying	☐	☐	☐	
9.Restlessness	☐	☐	☐	
10.Sense of loneliness	☐	☐	☐	
11.Sense of distress	☐	☐	☐	
12.Sense of loss	☐	☐	☐	
13.Inability to control emotions	☐	☐	☐	
14.Reduced self-confidence	☐	☐	☐	
15.Fear of cancer or treatment	☐	☐	☐	
16.Feeling low	☐	☐	☐	
17.Sense of desperation	☐	☐	☐	
Social problems subscale				
18.Feel uncomfortable discussing illness with others	☐	☐	☐	
19.Lack of social support	☐	☐	☐	
20.Financial problems	☐	☐	☐	
21.Would you like to discuss these problems with someone?	☐	☐	☐	
Sexual problems subscale				
22.Intimacy problems with partner	☐	☐	☐	☐
23.Less interest in sex	☐	☐	☐	☐
24.Would you like to discuss these problems with someone?	☐	☐	☐	☐

N/A: Not applicable
